# Effect of the eQuality Health Bwindi Scheme on Utilization of Health Services at Bwindi Community Hospital in Uganda

**DOI:** 10.3389/fpubh.2019.00071

**Published:** 2019-04-04

**Authors:** Doreen Birungi Agasha, Birungi Mutahunga R. Edwin, Sebastian Olikira Baine

**Affiliations:** ^1^Hospice Africa Uganda, Kampala, Uganda; ^2^Church of Uganda, Bwindi Community Hospital, Kanungu, Uganda; ^3^School of Public Health, College of Health Sciences, Makerere University, Kampala, Uganda

**Keywords:** eQuality, health insurance, community-based health insurance, access and utilization of health services, premium, copayment, understanding health insurance concept

## Abstract

**Introduction:** eQuality Health Bwindi (eQHB), a Community Based Health Insurance (CBHI) scheme was launched in March 2010 with the aim of generating income to maintain high quality care as well as increasing access to and utilization of health services at Bwindi Community Hospital (BCH). The main objective of this study was to explore evidence showing that eQHB scheme affected access and utilization of health services at BCH. The evidence generated would be used to inform decision making, policy and scale up of the scheme.

**Methods and Materials:** This study applied qualitative and quantitative research methods. It involved a review of hospital records for the period July 2009–June 2014, a survey of 272 households, four focus group discussions, and six key informant interviews. Both quantitative and qualitative analysis techniques were applied for the analysis.

**Results:** Outpatient attendance, inpatient admissions, and deliveries at the hospital increased by 65, 73, and 27%, respectively between FY 2009/10 and FY 2012/13. Utilization of health services by sick children from insured participants was greater than that of the uninsured members of the community (*p*-value = 0.0038). BCH services became more affordable. However, opting out of the scheme at a later stage in the review period was attributed to rising unaffordable premiums and co-payments. Failure to afford scheme membership, residing far from BCH and limited understanding of health insurance led to reduced BCH service utilization.

**Conclusions:** eQHB has potential to increase access and utilization of health services at BCH. The challenges are; limited understanding of the concept of health insurance and unaffordable premiums and co-payments set to enable provision of high quality services.

**Recommendations:** Based on these findings, intensified community sensitization on health insurance, establishment of satellite health facilities by BCH to bring services closer to members and transformation of eQHB to a savings/credit society in order to grow savings and subsequently reduce premiums are recommended. Government of Uganda should engage CBHIs countrywide to discuss achievement of UHC and establishment of a national health insurance scheme. A further study to guide setting of affordable premiums and copayments for eQHB is also recommended.

## Introduction

Health insurance has been perceived to enable access and utilization of good quality health services, and to provide protection against catastrophic health expenditure (1). Evidence shows that health insurance improves utilization of and accessibility to health services (1, 2), and diminishes catastrophic health expenditure among insured households (3). In addition, availability of essential drugs improved at health facilities operating CBHI schemes (4).

Health insurance has been applied in some settings, such as Europe to enable universal healthcare. Access to healthcare in Europe is achieved either through direct taxation or joining a health insurance scheme with contributions based on income. Access to healthcare in low and middle income countries is, in addition to several other challenges, hampered by high prices for medicines. In some countries, medicines cost 60–70% of total healthcare expenditure. This has potential to be catastrophic for the family (5). Efforts toward Universal Health Coverage in South Africa include improving access to medicines by controlling the prices and improving the supply chain (6).

The majority of countries in Sub Saharan Africa have implemented Community-based Health Insurance (CBHI) schemes, some on large scale, such as Ghana (7) whereby communities are actively engaged in resource mobilization and management (8). In a study on the factors influencing the performance of community-based health insurance (CBHI) on the health financing sub-functions, information on 85 CBHIs across Africa was reviewed. It was found that enrollment was below 10% for East and southern Africa and Rwanda while for west and central; Africa it ranged from 8 to 82%. In Senegal, enrollment ranged from 37 to 90%. The authors noted that most of the schemes needed more time to develop but despite this, low enrollment was attributed to challenges with affordability of premiums, varying levels of trust in the integrity of managers of the schemes, varying levels of trust in the competences of the scheme managers, how attractive the benefit package is and the quality of services provided under the schemes (9). Low enrollments, low premiums and high operational costs render them financially unsustainable and reliant on external support (4, 10, 11).

In Uganda, CBHI schemes started in the 1990s. Majority of them are affiliated to faith-based health facilities and are all coordinated by Uganda Community Based Health Financing Association (11, 12). eQuality Health Bwindi (eQHB) Scheme is a CBHI scheme implemented by Bwindi Community Hospital (BCH) since March 2010. The goal of the eQHB scheme is to increase community access to health care services at BCH. Bwindi Community Hospital (BCH) is a not for profit, faith based hospital that serves the community of Kayonza Subcounty and surrounding areas in Kanungu district. The Hospital offers a wide range of services including outpatient care, dental care, inpatient care, surgery, maternal and child health, pharmacy, radiological investigations and an effective timely referral system.

According to the Uganda National Household Survey 2016/17 (13), the main source of income for rural areas is crop farming which is seasonal followed by wage income. The average monthly wage income in rural areas is UGX 303,000 (equivalent to USD 84.2). This translates to an average daily wage of UGX 12,120 (equivalent to USD 3.4). Prior to the eQHB scheme, the outpatient service alone could cost between UGX 10,000 (USD 2.7) to UGX 30,000 (USD 8.1) excluding investigations and some expensive but necessary drugs. This cost would go much higher if one needed admission. So, patients at the hospital would either pay cash or in kind (e.g., livestock). Those that had nothing to pay were requested to commit to a debt that they would clear over time however most of them opted not to come to BCH for services. Within Kayonza subcounty where BCH is situated, there is a Government health facility that is at level three, with no Emergency obstetric care. Women in labor who could not afford BCH services would travel for 3 h on a nasty road to a government hospital where they could be helped adequately. BCH sought an equitable health financing method that patients could afford and at the same time the hospital could continue to provide high quality and wide coverage services without causing households to suffer catastrophic health expenditure because of paying for needed care. The idea of a community based health insurance scheme, eQHB scheme was born out of this background.

eQHB is based on the principle of risk sharing by pooling and rides on an already established tradition of burial societies. There are 182 registered active burial societies with wide coverage of over 95% of the local population. At least 80% of the members of the burial society are required to have paid premium to be eligible members of the scheme. On registration, all biographical information e.g., finger prints are entered into the eQHB database which keeps track of members' premiums, health records, service utilization and costs of treatment. eQHB scheme covers 38% of the targeted population (three subcounties of Kanungu district). Health care costs for children aged 5 years and below were initially catered for under the Child Health Access Project till June 2013 when the project closed and households took over responsibility to enroll their children into eQHB scheme. The annual premium is UGX 10,200 (equivalent to USD 2.8) per person above 5 years and UGX 6,000 (equivalent to USD 1.6) per child below 5 years. Insured clients make a co-payment of UGX 2,000 (less than USD1) for every hospital visit and can access outpatient care, dental care, inpatient care, surgery, maternal, and child health including medicines at BCH. eQHB scheme does not cover members referred to other health facilities for further management. The scheme members are allowed to pay quarterly premiums when they are unable to raise the total annual fee.

Funds generated by premiums and copayments meet about half the costs of care for members and the scheme's administration The other half is subsidized by donations from Development Partners and individuals interested in the hospital welfare (Bwindi Community Hospital annual report 2013/14). The impact of eQHB scheme has not been documented. It is not known whether eQHB scheme increased access and utilization of health services at BCH.

The objective of this study was to explore evidence showing that eQHB scheme affected access and utilization of health services at BCH. The hospital considered the scheme as a favorable financing mechanism that would enable the community in its catchment area use the hospital services in a sustainable way. Since its inception, there is no documented evidence of the changes in hospital utilization and so this study sought to explore the effects of the scheme on hospital utilization.

## Methods

### Study Design

This was a cross sectional study that used qualitative and quantitative research methods. It was conducted in Kayonza subcounty, which has a population of 34,021 people and 6,752 households. Membership of the eQHB scheme was 60% (20,433) of the population.

### Study Population

The study population included heads of insured and non-insured households, two hospital staff, two staff of eQHB, two community members, and outpatients, inpatients and maternity service records during the period July 2009 to June 2014.

### Sampling Procedure

In this study, the unit of sampling was a household. The minimum sample size of households in each group was obtained by using the Fleiss formula for determining the minimum sample size for a two proportion cross sectional study (14).

N_Fleiss_ = [Z_α/2_√(r + 1)(p)(1 – p) + Z_β_√rP_0_(1 – P_0_) + P_1_(1 – P_1_)]^2^/r(P_0_ – P_1_)^2^ where Z_α/2_ = 1.96 and Z_β_ = 0.84 and *p* = (P_0_ + rP_1_)/(r + 1). We used estimates of P_0_ and P_1_ taken from a study done in Ghana which assessed the effect of the country's national health insurance scheme on health care utilization (15). In this study, 90.5% of the insured population used formal health care when they were sick compared to 75.5% of the non-insured population.

So N_Fleiss_ = 106 respondents. In consideration of non-response estimated at 10%, the minimum sample size required to obtain a significant difference in proportions between the insured and the non-insured at a 5% level of significance and a power of 80% was 116 insured and 116 non-insured respondents, thus a total of 232.

### Inclusion Criteria

The study sample included selected households that consented by signature or thumbprint to participate in the study.

### Exclusion Criteria

Heads of the households who did not consent to participate in the study or where there was no eligible person to give informed consent.

### Data Collection

#### Quantitative Data

Quantitative data were collected on OPD attendance, hospital admissions, and deliveries during the financial years 2009/10–2013/14. Data were also collected on age, sex, marital status, level of education, occupation of household head, household sizes, number of children under 5 years of age in a household, monthly household income, distance of residence from BCH, utilization of BCH services, and membership and trends. Data was collected on distance to facilities as this is known to affect health service utilization (including medicines) as seen in Namibia (16).

#### Qualitative Data

Qualitative data were obtained on dynamics within eQHB scheme and opinions on the eQHB scheme using four focus group discussions: two insured (male and female) and two non-insured (male and female); and six key informants (two hospital staff (the Executive Director and a clinical officer), two management staff of eQHB scheme, and two community leaders). Data collection **t**ools were pretested in a neighboring Sub-county to ensure validity and consistency of the data collection tools prior to using them in the main study.

### Study Variables

The dependent variables were access, utilization of health services at BCH and being member of eQHB scheme. The independent variables were head of household characteristics, household size, household income, social economic status, perception of severity of most recent illness, nature of illness suffered, distance from BCH, and opinions about the scheme.

### Data Management and Analysis

Quantitative data was reviewed for completeness and consistency. It was coded, entered into Epi data software v3.1, cleaned and exported to SPSS version 14 for analysis. All qualitative data including transcribed data from recordings and notes from the interviews and discussions were translated into English. The data was analyzed using thematic analysis.

## Results

### Demographic Characteristics

A total of 272 households were included in this study. Of the 272 respondents, only 125 (46.1%) were insured and the rest had dropped out or never been members of eQHB scheme (Table 1). Majority of the respondents were male (83.5%), aged 36 and more years (59.6%) and married (89%) (Table 2).

**Table 1:** Distribution of respondents by parish in Kayonza Subcounty.

**Parish**	**Total population**	**Number of households**	**Number of respondents (household heads interviewed)**		
			**Insured**	**Non-insured**	**Total**
Bujengwe	5,988	1,165	26	59	85
Karangara	3,926	756	9	21	30
Kyeshero	8,647	1,645	24	25	49
Mukono	7,890	1,613	31	17	48
Ntungamo	7,570	1,573	35	25	60
Total	34,021	6,752	125	147	272

**Table 2:** Characteristics of the respondents.

**Characteristic**	**Frequency (*n* = 272)**	**Percentage**
**AGE CATEGORY OF THE HOUSEHOLD HEAD**		
18–25 years	19	7
26–35 years	91	33.5
More than 36 years	162	59.6
**SEX OF THE HOUSEHOLD HEAD**		
Male	227	83.5
Female	45	16.5
**MARITAL STATUS OF THE HOUSEHOLD HEAD**		
Single	19	7
Married	242	89
Divorced	5	1.8
Separated	6	2.2
**LEVEL OF EDUCATION OF THE HOUSEHOLD HEAD**		
None	33	12.1
Primary	180	66.2
Senior four (O-level)	29	10.7
Senior six (A Level)	8	2.9
Tertiary	22	8.1
**OCCUPATION OF THE HOUSEHOLD HEAD**		
None	15	5.5
Self employed	118	43.4
Formal employment	26	9.6
Farmer	113	41.5
**OWNERSHIP OF LAND**		
Yes	249	91.5
No	23	8.5
**OWNERSHIP OF LIVESTOCK**		
Yes	196	72.1
No	76	27.9
**ESTIMATED MONTHLY INCOME**		
Less than UGX 50,000/month	64	23.5
UGX 51,000–100,000/month	116	42.6
More than UGX 100,000/month	92	33.8
**ESTIMATED ANNUAL EXPENDITURE ON HEALTH**		
Less than UGX 50,000/year	86	31.6
UGX 51,000–100,000/year	83	30.5
More than UGX 100,000/year	103	37.9
**DISTANCE FROM BCH**		
Less than 1 km	28	10.3
1–5 km	66	24.3
More than 5 km	178	65.4

The respondents who came to BCH for their own treatment were 172 (Table 3). Among them, more of the insured household heads (55.4%) were peasant farmers while more of the non-insured were self-employed, *p*-value = 0.018 (Table 4). There were no significant differences between the insured and the non-insured in regard to marital status, level of education and household size (Table 4). There were no significant differences in annual household expenditure on health, average household daily expenditure or possession of assets, such as land, livestock among others between the insured and the non-insured (Table 5).

**Table 3:** Respondents that recall utilizing BCH services.

**Profile**	**Insured**	**Non-insured**	**Total**	**Profile**	**Insured**	**Non-insured**	**Total**
**Sought BCH care for self**				**Sought BCH care for a sick child**			
Yes	83 (66.4%)	89 (60.5%)	172	Yes	67 (53.6%)	55 (37.4%)	122
No	42 (33.6%)	58 (39.5%)	100	No	58 (46.4%)	92 (62.6%)	150
Total	125	147	272	Total	125	147	272

**Table 4:** Household characteristics for the respondents that visited BCH for self.

**Profile**	**Insured (*n* = 83)**	**Non-insured (*n* = 89)**	***p*-Value**	**OR (95% CI)**
**AGE CATEGORY**				
18–25 years	4 (4.8%)	5 (5.6%)	0.033^*^	
26–35 years	22 (26.5%)	40 (44.9%)		
≥36 years	57 (68.7%)	44 (49.4%)		
**SEX**				
Male	67 (80.7%)	74 (83.1%)	0.680	0.849 (0.390–1.848)
Female	16 (19.3%)	15 (16.9%)		
**MARITAL STATUS**				
Single	5 (6.0%)	1 (1.1%)	0.230	
Married	75 (90.4%)	84 (94.4%)		
Divorced	1 (1.2%)	3 (3.4%)		
Separated	2 (2.4%)	1 (1.1%)		
**LEVEL OF EDUCATION**				
None	5 (6.0%)	11 (12.4%)	0.358	
Primary	59 (71.1%)	59 (66.3%)		
Senior four (O-level)	7 (8.4%)	10 (11.2%)		
Senior six (A-level)	4 (4.8%)	1 (1.1%)		
Tertiary	8 (9.6%)	8 (9.0%)		
**OCCUPATION**				
None	2 (2.4%)	4 (4.5%)	0.018^*^	
Self employed	25 (30.1%)	46 (51.7%)		
Formal employment	10 (12.0%)	9 (10.1%)		
Farmer	46 (55.4%)	30 (33.7%)		
**HOUSEHOLD SIZE**				
Up to 4 people	28 (33.7%)	38 (42.7%)	0.227	0.683 (0.368–1.269)
More than 4 people	55 (66.3%)	51 (57.3%)		

* *p-value < 0.05*.

**Table 5:** Socio-economic status and income of the respondents that visited BCH for self.

**Profile**	**Insured (*n* = 83)**	**Non-insured (*n* = 89)**	***p*-Value**	**OR (95% CI)**
**ESTIMATED MONTHLY INCOME**				
Less than UGX 50,000	18 (21.7%)	18 (20.2%)	0.538	
UGX 51,000–100,000	39 (47.0%)	36 (40.4%)		
More than UGX 100,000	26 (31.3%)	35 (39.3%)		
**AVERAGE HOUSEHOLD DAILY EXPENDITURE**				
Less than UGX 2,500	11 (13.3%)	17 (19.1%)	0.583	
Between UGX 2,500 and UGX 5,000	37 (44.6%)	37 (41.6%)		
More than UGX 5,000	35 (42.2%)	35 (39.3%)		
**LAND OWNERSHIP**				
Yes	76 (91.6%)	83 (93.3%)	0.675	0.785 (0.253–2.439)
No	7 (8.4%)	6 (6.7%)		
**OWNERSHIP OF CURRENT RESIDENCE**				
Owned	72 (86.7%)	82 (92.1%)	0.503	
Rented	9 (10.8%)	6 (6.7%)		
Free accommodation provided	2 (2.4%)	1 (1.1%)		
**OWNERSHIP OF LIVESTOCK**				
Yes	58 (69.9%)	69 (77.5%)	0.254	0.672 (0.339–1.332)
No	25 (30.1%)	20 (22.5%)		
**OWNERSHIP OF A BICYCLE, MOTORCYCLE OR CAR**				
Yes	17 (20.5%)	15 (16.9%)	0.541	1.271(0.589–2.743)
No	66 (79.5%)	74 (83.1%)		
**ESTIMATED ANNUAL EXPENDITURE ON HEALTH FOR THE HOUSEHOLD**				
Less than 50,000/=	23 (27.7%)	23 (25.8%)	0.169	
51,000/ = – 100,000/=	21 (25.3%)	34 (38.2%)		
More than 100,000/=	39 (47.0%)	32 (36.0%)		

The respondents who brought a sick child to BCH for treatment in the last 6 months were 122 (Table 3). Even among these, more of the insured household heads (55.2%) were peasant farmers while more of the non-insured (58.2%) were self-employed, *p*-value = 0.005 (Table 6). There were no significant differences between the insured and the non-insured in regard to marital status, level of education, household size and number of children 5 years and below in the household (Table 6). Approximately 42% of the insured earned a range of UGX 51,000–100,000 per month while more than half (56.4%) of the non-insured earned more than UGX 100,000 per month, *p*-value = 0.020 (Table 7).

**Table 6:** Characteristics of households that took a sick child to BCH in the past 6 months.

**Profile**	**Insured (*n* = 67)**	**Non-insured (*n* = 55)**	***p*-Value**	**OR (95% CI)**
**AGE CATEGORY OF HOUSEHOLD HEAD**				
18–25 years	2 (3.0%)	3 (5.5%)	0.369	
26–35 years	18 (26.9%)	20 (36.4%)		
≥36 years	47 (70.1%)	32 (58.2%)		
**MARITAL STATUS OF HOUSEHOLD HEAD**				
Single	4 (6.0%)	0 (0%)	0.107	
Married	60 (89.6%)	55 (100%)		
Divorced	1 (1.5%)	0 (0%)		
Separated	2 (3.0%)	0 (0%)		
**LEVEL OF EDUCATION OF HOUSEHOLD HEAD**				
None	5 (7.5%)	3 (5.5%)	0.365	
Primary	49 (73.1%)	39 (70.9%)		
Senior four (O-level)	4 (6.0%)	7 (12.7%)		
Senior six (A-level)	3 (4.5%)	0 (0%)		
Tertiary	6 (9.0%)	6 (10.9%)		
**OCCUPATION OF HOUSEHOLD HEAD**				
None	2 (3.0%)	0 (0%)	0.005^*^	
Self employed	19 (28.4%)	32 (58.2%)		
Formal employment	9 (13.4%)	7 (12.7%)		
Farmer	37 (55.2%)	16 (29.1%)		
**HOUSEHOLD SIZE**				
Up to 4 people	17 (25.4%)	17 (30.9%)	0.497	0.760 (0.344–1.681)
More than 4 people	50 (74.6%)	38 (69.1%)		
**NUMBER OF CHILDREN** **≤5 YEARS IN THE HOUSEHOLD**				
None	22 (32.8%)	12 (21.8%)	0.319	
Two or less	39 (58.2%)	35 (63.6%)		
Above 2	6 (9.0%)	8 (14.5%)		

**Table 7:** Socio-economic status of the respondents who took a sick child to BCH.

**Profile**	**Insured (*n* = 67)**	**Non-insured (*n* = 55)**	***p*-Value**	**OR (95% CI)**
**ESTIMATED MONTHLY INCOME**				
Less than UGX 50,000	17 (25.4%)	6 (10.9%)	0.020^*^	
UGX 51,000–UGX 100,000	28 (41.8%)	18 (32.7%)		
More than UGX 100,000	22 (32.8%)	31 (56.4%)		
**AVERAGE HOUSEHOLD DAILY EXPENDITURE**				
Less than UGX 2,500	11 (16.4%)	5 (9.1%)	0.411	
Between UGX 2,500 and UGX 5,000	29 (43.3%)	23 (41.8%)		
More than UGX 5,000	27 (40.3%)	27 (49.1%)		
**LAND OWNERSHIP**				
Yes	63(94.0%)	51 (92.7%)	0.772	1.235 (0.294–5.184)
No	4 (6.0%)	4 (7.3%)		
**OWNERSHIP OF CURRENT RESIDENCE**				
Owned	61 (91.0%)	51 (92.7%)	0.826	
Rented	4 (6.0%)	2 (3.6%)		
Free accommodation provided	2 (3.0%)	2 (3.6%)		
**OWNERSHIP OF LIVESTOCK**				
Yes	47 (70.1%)	44 (80.0%)	0.214	0.588 (0.253–1.365)
No	20 (29.9%)	11 (20.0%)		
**OWNERSHIP OF A BICYCLE, MOTORCYCLE OR CAR**				
Yes	13 (19.4%)	13 (23.6%)	0.570	0.778 (0.326–1.853)
No	54 (80.6%)	42 (76.4%)		
**ESTIMATED ANNUAL EXPENDITURE ON HEALTH FOR THE HOUSEHOLD**				
Less than 50,000/=	17 (25.4%)	9 (16.4%)	0.238	
51,000/ = – 100,000/=	19 (28.4%)	23 (41.8%)		
More than 100,000/=	31 (46.3%)	23 (41.8%)		

* *p-value < 0.05*.

### Membership in eQHB Scheme

All the key informants reported the hospital had received more clients since the launch of eQHB. Membership grew to 29,000 by the end of 2013 from the time of its launch in March 2010. The insured participants reported that since joining the eQHB scheme they appreciated the concept and others were encouraged to join by people with positive experiences.

The households loved the scheme because it was affordable and enabled them to access quality care at affordable prices. The most attractive aspect of the scheme was the ability to get treatment at as low as UGX 2,000 (less than USD1) only and emergency care even when disposable income is not available. The insured members obtained treatment at BCH fast and were not anxious about payment as was the case before the scheme started.

Many of the non-insured respondents had dropped out of eQHB scheme partly because premiums were increased which affected mostly large families and partly because households residing far from BCH were not routinely using services at BCH and benefiting from the scheme due to high transport costs. They initially enrolled in the scheme in order to access emergency care and sought care for minor ailments from nearby affordable health providers.

### Utilization of Health Services

**Figure 1:**
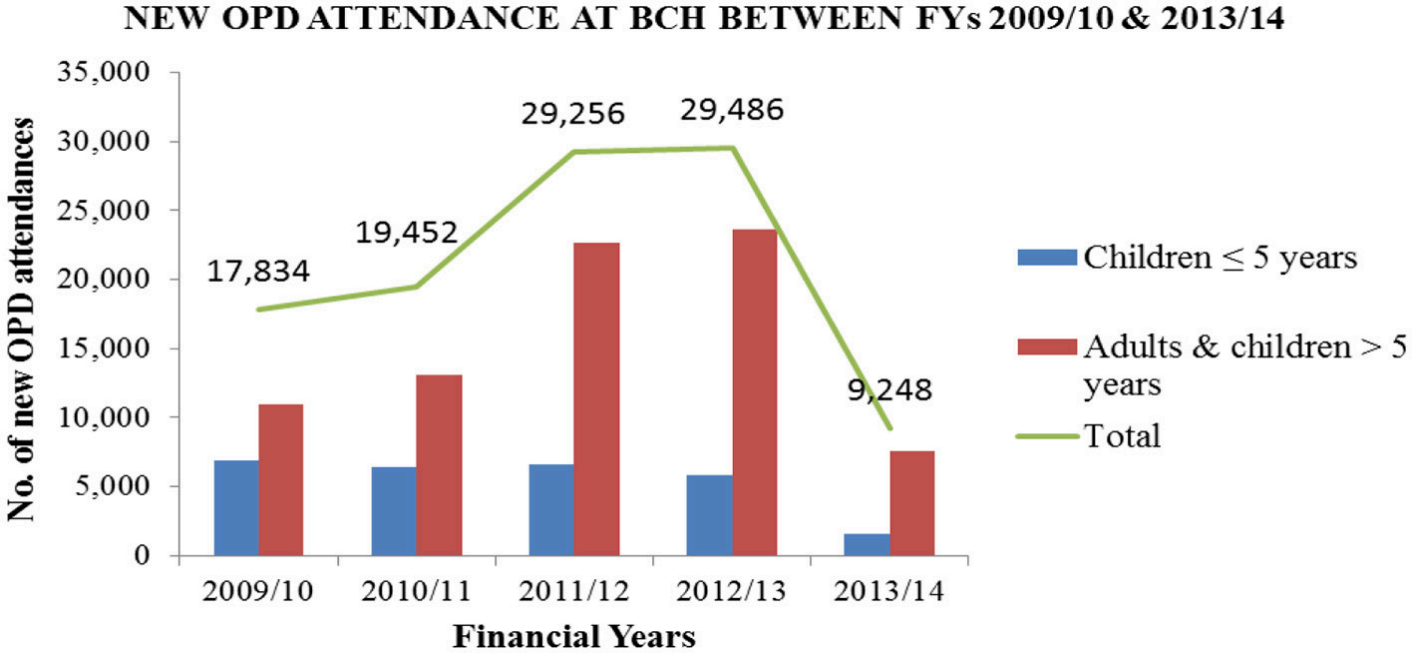
Utilization of OPD services at BCH for the financial years 2009/10 to 2013/14.

A review of outpatient department (OPD) records showed an increase in outpatient attendance from 17,834 patients in FY 2009/10 to 29,486 patients in FY 2012/13. OPD attendance dropped in 2013/2014 to 9,248 patients. This was attributed to the revision of the premiums required in order to be a member of the eQHB scheme and closure of the Child Health Access Project that subsidized care for children below 5 years. The trend of utilization of outpatient health services at BCH is shown in Figure 1.

**Figure 2:**
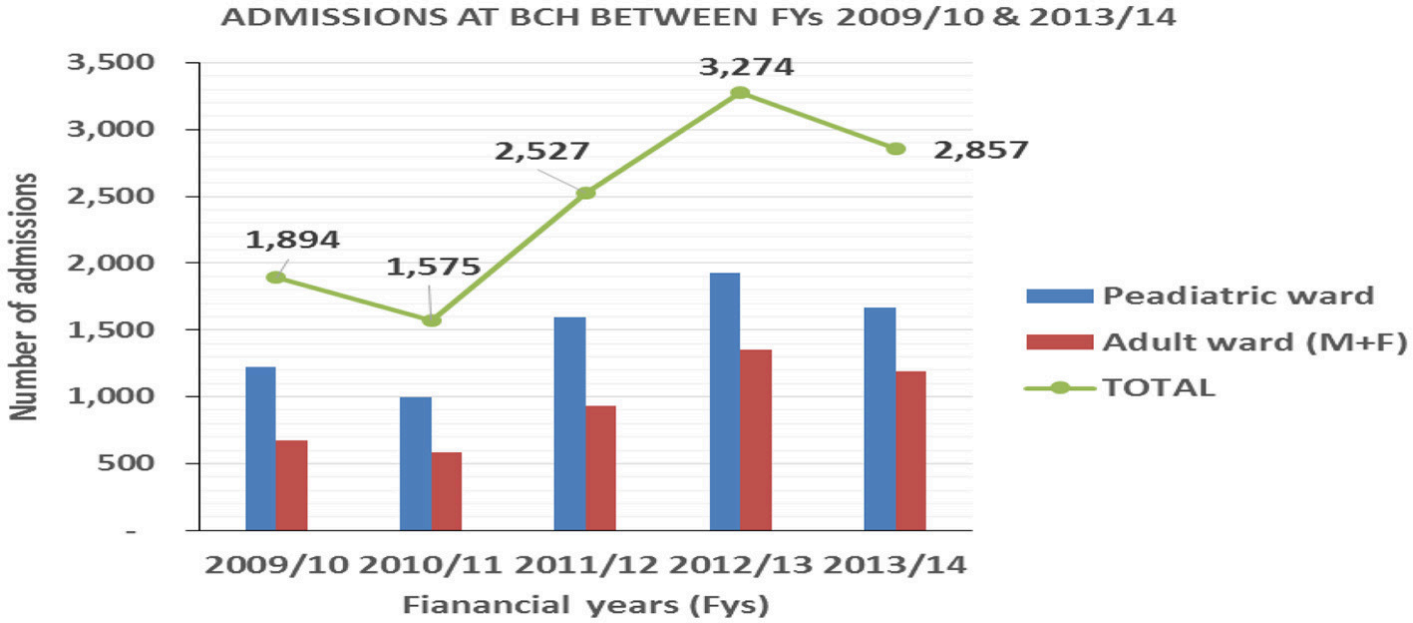
Admissions at BCH for the financial years 2009/10 to 2013/14.

Annual admissions increased from 1,894 patients in FY 2009/10 to 3,274 patients in FY 2012/13 followed by a decrease in FY2013/14 as shown in Figure 2. This was attributed to revision of the premiums, closure of the Child Health Access Project and parents failing to take full responsibility of their children's premiums and copayments required by eQHB scheme.

**Figure 3:**
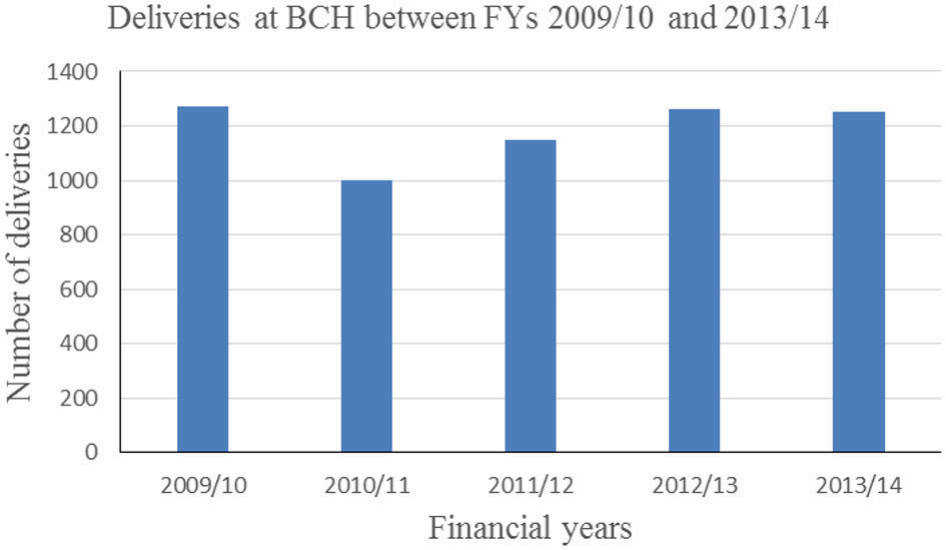
Deliveries at BCH for the financial years 2009/10 to 2013/14.

Annual deliveries increased from 1,250 deliveries in 2009/10 to 1,264 deliveries in 2012/13 and a slight decrease to 1,250 in 2013/14 as shown in Figure 3. Hospital deliveries in 2009/10 were subsidized by a voucher project that ended 2010. There has been an increase in deliveries from 2011/12 onwards.

Based on BCH records, utilization of services increased by 65, 73, and 27% in OPD, IPD and annual deliveries, respectively after the launch of eQHB scheme in 2010.

Data from the household survey showed that the proportion of insured household heads who sought care at BCH for self was 66.4% while it was 60.5% for the non-insured households [z = 0.99; P (Z ≤ z) = 0.8389], a non-significant finding with a *p*-value = 0.1611. Among those that sought BCH care for a sick child, the proportion of the insured was 53.6% while the proportion of the non-insured was 37.4; [z = 2.67, P (Z ≤ z) = 0.9962] a significant finding with a *p*-value = 0.0038. The proportion of the insured who used the hospital for a sick child was greater than the proportion of the non-insured who used the hospital for the same purpose unlike those who use the hospital for their own health where the insured and the non-insured did not differ significantly (Table 3).

In the FGDs, the insured participants reported an increase in the utilization of health services at BCH between 2010 and 2014. They reported that they seek treatment early before a condition worsens. A participant from FGD 3 said,

“*Before the eQHB scheme, you would fear to go to the hospital because of cost. But now, you can go even when it is just a headache and not wait for when you are badly off*.”

Clients accessed reliable quality services and received adequate medication for the stipulated period of time. One of the participants in FGD 2 said,

“*I am grateful. Before the eQHB scheme, we would get few drugs and most times you wouldn't get better. But these days, they give you enough medicine and you actually recover.”*

eQHB scheme increased health services utilization at BCH and was beneficial to members. It eased access to healthcare in emergency situations and reduced the risk of catastrophic health expenditure by households.

Non-insured participants reported a reduced utilization of health services at BCH between 2010 and 2014. They used alternative sources of health care, such as government health facilities, clinics, traditional medicine among others. BCH was only considered a place to go in emergency situations especially sick children.

### Distance From the Health Facility

Among those that used BCH services for their own care, majority (67.4%) of non-insured resided near a government health facility while the majority (51.8%) of the insured did not reside near a Government health facility, *p*-value = 0.011 (Table 8). The Government health facility offers free services, an option that could result in disinterest in the scheme. Among those who sought BCH care for a sick child, majority (55.2%) of the insured lived within 5 km of BCH while majority (74.5%) of the non-insured lived more than 5 km away from BCH, *p*-value = 0.003, Table 9 showing that distance from the facility providing health insurance may affect enrollment into the scheme and thus utilization of services. The insured and the non-insured users of BCH services differed significantly in their geographical access to BCH and the government health facilities.

**Table 8:** Geographical access to BCH among the respondents that visited BCH for self.

**Profile**	**Insured (*n* = 83)**	**Non-insured (*n* = 89)**	***p*-Value**
**DISTANCE OF HOUSEHOLD FROM THE NEAREST HEALTH FACILITY**			
Less than 1 km	37 (44.6%)	30 (33.7%)	0.154
1–5 km	39 (47.0%)	44 (49.4%)	
More than 5 km	7 (8.4%)	15 (16.9%)	
**DISTANCE OF BCH FROM HOUSEHOLD**			
<1 km	15 (18.1%)	6 (6.7%)	0.000^*^
1–5 km	33 (39.8%)	19 (21.3%)	
>5 km	35 (42.2%)	64 (71.9%)	
**HAVING THE NEAREST HF AS A GOVERNMENT HF**			
Yes	40 (48.2%)	60 (67.4%)	0.011^*^
No	43 (51.8%)	29 (32.6%)	

* *p-value < 0.05*.

**Table 9:** Geographical access to BCH among respondents who took a sick child to BCH.

**Profile**	**Insured (*n* = 67)**	**Non-insured (*n* = 55)**	***p*-Value**
**DISTANCE OF HOUSEHOLD FROM THE NEAREST HEALTH FACILITY**			
Less than 1 km	27 (40.3%)	17 (30.9%)	0.446
1–5 km	33 (49.3%)	29 (52.7%)	
More than 5 km	7 (10.4%)	9 (16.4%)	
**DISTANCE OF BCH FROM HOUSEHOLD**			
<1 km	9 (13.4%)	5 (9.1%)	0.003^*^
1–5 km	28 (41.8%)	9 (16.4%)	
>5 km	30 (44.8%)	41 (74.5%)	
**HAVING THE NEAREST HF AS A GOVERNMENT HF**			
Yes	33 (49.3%)	39 (70.9%)	0.016^*^
No	34 (50.7%)	16 (29.1%)	

* *p-value < 0.05*.

Among those that sought BCH care for themselves, majority (48.8%) of the insured users reported non-severe illness while majority (42.5%) of the non-insured users reported moderately severe illness, *p*-value = 0.023 as shown in Table 10. Among those that sought BCH care for a sick child, there was no significant difference in severity of illness (Table 11).

**Table 10:** Severity of illness among the respondents that visited BCH for self.

**Profile**	**Insured (*n* = 83)**	**Non-insured (*n* = 89)**	***p*-Value**
**SEVERITY OF THE ILLNESS OF THE HOUSEHOLD HEAD**			
Not severe	40 (48.8%)	29 (33.3%)	^*^0.023
Moderately severe	34 (41.5%)	37 (42.5%)	
Very severe	8 (9.8%)	21 (24.1%)	
**ADMISSION OF THE HOUSEHOLD HEAD**			
Yes	17 (20.5%)	23 (26.4%)	0.360
No	66 (79.5%)	64 (73.6%)	

* *p-value < 0.05*.

**Table 11:** Severity of illness of the children of the respondents.

**Profile**			***p*-Value**
**SEVERITY OF THE ILLNESS OF THE CHILD TAKEN TO BCH IN THE PAST 6 MONTHS**			
	**Insured (*****n*** **=** **66)**	**Non-insured (*****n*** **=** **53)**	
Not severe	26 (39.4%)	20 (37.7%)	0.640
Moderately severe	25 (37.9%)	24 (45.3%)	
Very severe	15 (22.7%)	9 (17.0%)	
**ADMISSION OF THE CHILD**			
	**Insured (*****n*** **=** **67)**	**Non-insured (*****n*** **=** **53)**	
Yes	25 (37.3%)	12 (22.6%)	0.084
No	42 (62.7%)	41 (77.4%)	

In the multivariate analysis, all respondents, 195 (71.7%) who used BCH services for self and those who took a sick child to BCH were combined. In this analysis variables that apply to both groups of users and only those with a *p*-value <0.2 were included in the logistic regression shown in Table 12. The significant finding was that respondents who used BCH services and lived more than 5 km away from BCH were 4.8 times more likely to be insured; *p*-value 0.03.

**Table 12:** Results of the Multivariable analysis comparing users of BCH on insurance status.

**Category**	**Frequency**	**Adjusted OR (95% CI)**	***p*-Value**
**OCCUPATION OF THE HOUSEHOLD HEAD**			
None	6	1.00	0.1
Self employed	81	0.687 (0.093–5.087)	0.7
Formal employment	20	0.246 (0.027–2.215)	0.2
Farmer	88	0.286 (0.040–2.056)	0.2
**MARITAL STATUS OF THE HOUSEHOLD HEAD**			
Single	7	1.00	0.5
Married	181	4.579 (0.517–40.563)	0.2
Divorced	4	10.704 (0.383–299.357)	0.2
Separated	3	2.922 (0.100–85.126)	0.5
**ANNUAL HOUSEHOLD EXPENDITURE ON HEALTH**			
Less than UGX 50,000/year	55	1.00	0.6
UGX 51,000–100,000/year	63	1.465 (0.629–3.411)	0.4
More than UGX 100,000/year	77	1.035 (0.437–2.453)	0.9
**ESTIMATED MONTHLY INCOME**			
Less than UGX 50,000/month	41	1.00	0.8
UGX 51,000–100,000/month	85	1.039 (0.435–2.482)	0.9
More than UGX 100,000/month	69	1.364 (0.499–3.732)	0.5
**DISTANCE OF THE HOUSEHOLD FROM BCH**			
Less than 1 km	23	1.00	0.03
1–5 km	57	1.055 (0.358–3.110)	0.9
More than 5 km	115	4.813 (1.1698–19.337)	0.03^*^
**IS YOUR NEAREST HEALTH FACILITY A GOVERNMENT**			
**HEALTH FACILITY**			
No	81	1.00	
Yes	114	0.450 (0.138–1.471)	0.2

* *p-value < 0.05*.

### Affordability of Health Services at BCH

The non-insured reported paying almost twice the amount that the insured pay for the same services at BCH. They therefore opted for alternative services which are more affordable but not as reliable as at BCH. They reported that often the condition worsened necessitating emergency care usually sough from BCH. The medical bill following this emergency treatment would be enormous and the user has to sell property to meet it. One of the participants from FGD 4 said,

“*I always fear the bill. I only go to BCH when I have no alternative. I would otherwise go to Kambuga hospital except that sometimes the services may not be available since the senior health workers like the doctors are always on and off.”*

Another participant said, “*Other facilities charge relatively low fees though services may not be as good*.”

A non-insured participant who had recently dropped out of the eQHB scheme expressed desire to join again in the near future.

“*I was charged a lot of money at Bwindi hospital when I fell sick; yet, people in eQuality Health Bwindi scheme were charged less. So I decided to come back to the scheme also*,” said a participant from FGD 3.

### Attitude Toward eQHB

Both groups conceded that the scheme was beneficial especially in emergency situations where disposable income may not be readily available to meet the emergency care expenditures. The fear of unplanned sale of assets for medical care was reduced among the insured as they no longer sold their household assets or borrowed money from friends and money lenders to raise money to pay medical bills. They didn't have to forego basic needs at home as it was before the existence of *eQHB* scheme.

All the insured were uncomfortable with the new premium and copayments at BCH because they were high. Despite the provision for quarterly payment, households still felt the charges were high. They said they were struggling to stay in the scheme and some even considered leaving the scheme.

“*I dig for other people to get money. I also dig in my own garden to get food and what to sell so that I can buy salt, etc. I will fail to be in eQHB if premium is increased. Increase from UGX 2000 to UGX 3400 was too much. They should not increase any more*,” said a participant from FGD4.

Hospital services' utilization decreased in OPD and inpatient care largely because of the reduction in scheme membership. By September 2014, scheme membership had dropped by 14% to about 25,000 members. Consultation with burial societies revealed that the decline in membership was attributed to increased premium and co-payments; and opting out of the scheme by those who live far away from the hospital.

Some of the households could not afford the new premiums and co-payments, and decided not to renew their membership. Other members found it costly in terms of transport to the hospital and opted out. Active burial society groups reduced by 11.7% between 2012 and 2014. One of the key informants said,

“*Every three months, members pay 2,550/* = *per person. It is collected by burial society group leaders. Some groups leaders became dormant when premiums were revised. There were 206 active burial groups registered in December 2012 which reduced to 182 in December 2014.”*

### Level of Understanding of the Concept of Insurance by the Community

The non-insured participants argued that paying for healthcare in advance was unnecessary. They questioned the need to pay for healthcare when one is well in anticipation of a time they would be unwell. They also disagreed with the notion of paying money to settle another member's medical bill. Burial society leaders were getting discouraged because some households were refusing to pay for the quarter or to come to meetings. Burial society leaders said,

“*People have been going to BCH a lot because of eQHB. Increasing the money for eQHB has caused many people to use other health centres because they cannot afford to go to BCH even though it has very good services,”*

Three key informants; two from the hospital and one community leader expressed concern that the community members did not understand health insurance, how it operates and the benefits. In some instances, people spread negative propaganda claiming that the insured receive cheaper and poor quality drugs. This caused scheme members to drop out and impacted on utilization of health services at BCH. Key informants from the community said,

“*We still have people who refuse to join eQHB because they say they never fall sick and yet they never need to use the hospital services. They feel they have wasted their money if they take a year of paying membership. Such people were very quick to drop out of the scheme when the premiums were increased.”*

## Discussion

The results of the study showed an increase in BCH service utilization. Based on BCH records, utilization of services increased by 65, 73, and 27% in OPD, IPD and annual deliveries, respectively after the launch of eQHB scheme in 2010.

The increase in service utilization could be attributed to affordable health care at the hospital made possible by the eQHB scheme. Patients had reduced inhibition to seek care partly because they were not required to have cash at hand to pay for the services. Similar findings were reported by Ranabhat et al. (2) who reported a significant increase in health service utilization after studying six Government financed and six Co-operative financed CBHIs in Nepal, India. In this study, the health service utilization rates went up to 107% for Government financed schemes and 137% for Co-operative financed schemes, seemingly higher for the co-operative financed schemes however the difference is not statistically significant. Cheng and Chiang (17) reported similar findings in Taiwan where outpatient and inpatient care doubled as a result of Universal Health Insurance. In addition, the increase in service utilization could be attributed to increased access and community awareness about the hospital services during efforts to promote the scheme. Rebahn (18) in his analysis of three models of health seeking behavior namely; The Health Belief Model by Rosenstock, Andersen's model and Young's choice making model concludes that the decision to seek health care is based on access, culture and social networks.

The decrease in service utilization at BCH in the financial year 2013/14 was attributed to increase in the premium and co-payments. This demonstrated unaffordability on the part of the households especially large households and subsequently low coverage. In explicit terms, people with 7 household members would pay on a quarterly basis UGX 17,850 (equivalent to USD 5) requiring the household to earn every month which is difficult for a majorly peasant community that earns seasonally. Ranabhat et al. (2) also reports similar findings for the twelve health insurance schemes studied where the new enrollments were low and the retention of those enrolled was low. In this study, decreased enrollment rates were attributed to uncertainty in financial viability, quality of care especially those under the government financed scheme who felt they were no different from those outside the scheme, long waiting time when seeking care and poor management skills of healthcare providers. Those under the government financed scheme also reported that they felt financial loss when they never fell sick and did not gain from the benefit package of the scheme, similar to findings in this study that contributed to drop out rates. Other factors related to the decreased utilization and coverage that have been reported in other studies are low understanding of health insurance, history of insurance in Africa, low coverage, and poor generation of resources (9). Also the design of the scheme seems to exclude large households although household size was not a significant difference between the insured and the non-insured in the bivariate analysis.

Residing far from the hospital negatively affected enrolment in eQHB scheme as noted by the drop out of members that felt they were far away from the hospital. This also affected utilization of BCH services because the transport costs were high. Those who used the hospital and lived more than 5 km away were 4.8 times more likely to be insured probably because they were compelled by the need to benefit from the scheme. The challenge with distance was that the cost of transport in the area was high and so accessing BCH could not be as regular as desired by the insured.

As countries make strides toward Universal Health Coverage, it is important that all populations are able to access healthcare without any household suffering catastrophic health expenditure (19, 20). This study alongside other studies as noted in the review by Ranabhat et al. (1) show that community based health insurance schemes have significant potential. In this study, we demonstrated that BCH which offers not for profit services was able to increase its service utilization rates using eQuality Health Bwindi scheme. However, enrollment in the schemes and retention of enrollees remains a significant challenge which if not addressed impedes efforts toward Universal Health Coverage.

## Limitations of the Study

1. It was not possible to accurately determine the insured and the non-insured population because insurance status is updated every quarter in the eQHB database but does not capture all individual patient records
2. It was difficult to obtain equal numbers of the insured and the non-insured however the sample size obtained was adequate to achieve the minimum power desired of 80%.
3. The study design used only provides information on associations observed regarding the hospital utilization and the CBHI scheme at a specific point in time.

## Conclusion

There was an increase in the utilization of BCH services after the launch of eQHB scheme in March 2010.

Residing far from the hospital negatively affected utilization of health services and enrolment in eQHB. This was attributed to high premium coupled with high transport costs to access the services.

The challenges to enrollment in the eQHB scheme were limited household understanding of the concept of health insurance, perceived high premiums and co-payments, and overcoming the negative history of insurance in the study area and associated negative propaganda.

## Recommendations

Intensify continuous community sensitization on the concept of health insurance to attract more subscribers and increase membership in the eQHB scheme and utilization of health services at the BCH.

Establish satellite clinics within the sub-county of Kayonza in order for services to get closer to users.

Operate eQHB scheme as a cooperative or savings and credit society. In this way, the scheme can grow savings which will be used to meet healthcare costs and credit services causing the scheme to be more acceptable to the community. Premiums will gradually reduce as savings increase.

A longitudinal study is proposed to establish ways in which premiums and copayments in a CBHI scheme can be increased without negatively affecting membership, quality and utilization of health services at Bwindi Community Hospital.

Health insurance has the potential to enhance utilization of health services and to cause significant achievements toward Universal Health Coverage. Therefore, Government of Uganda should bring together CBHIs from around the country and other players running health insurance schemes to discuss achievement of UHC and establishment of a national health insurance scheme. The discussions can also explore how to incorporate the already existing CBHIs into the national health insurance scheme.

## Data Availability

The datasets generated for this study are available on request to the corresponding author.

## Ethics Statement

This study was carried out in accordance with the recommendations of the “Makerere University Research Ethics Committee” with written informed consent from all subjects. All subjects gave written informed consent in accordance with the Declaration of Helsinki. The protocol was approved by the “Makerere University Research Ethics Committee.” Bwindi Community Hospital Management granted permission to access its database.

## Author Contributions

DA prepared the research proposal, conducted the research, and prepared the report. SB supervised the research at Makerere University College of Health Sciences, School of Public Health. The University IRB approved this research and he was responsible for ensuring that it was conducted within the ethical standards prescribed by the IRB. He also contributed significantly to interpretation of the findings and the report writing. BE is the Executive Director of Bwindi Community Hospital and he was instrumental in identifying the research area as part of the quality improvement agenda of the hospital. He ensured that the research was smoothly conducted at Bwindi Community Hospital. He provided approval for access to the patients records and ensured that the information obtained from the hospital for review was correct. He contributed significantly to the interpretation of the findings and the report writing.

### Conflict of Interest Statement

The authors declare that the research was conducted in the absence of any commercial or financial relationships that could be construed as a potential conflict of interest.
